# Seventy Years of Asthma in Italy: Age, Period and Cohort Effects on Incidence and Remission of Self-Reported Asthma from 1940 to 2010

**DOI:** 10.1371/journal.pone.0138570

**Published:** 2015-10-06

**Authors:** Giancarlo Pesce, Francesca Locatelli, Isa Cerveri, Massimiliano Bugiani, Pietro Pirina, Ane Johannessen, Simone Accordini, Maria Elisabetta Zanolin, Giuseppe Verlato, Roberto de Marco

**Affiliations:** 1 Unit of Epidemiology & Medical Statistics, Dept. of Public Health & Community Medicine, University of Verona, Verona, Italy; 2 Unit of Respiratory Diseases, IRCCS Policlinico San Matteo, University of Pavia, Pavia, Italy; 3 Unit of Respiratory Medicine, ASL TO-2, Turin, Italy; 4 Institute for Respiratory Diseases, University of Sassari, Sassari, Italy; 5 Centre for Clinical Research, Haukeland University Hospital, Bergen, Norway; Peking University, CHINA

## Abstract

**Background:**

It is well known that asthma prevalence has been increasing all over the world in the last decades. However, few data are available on temporal trends of incidence and remission of asthma.

**Objective:**

To evaluate the rates of asthma incidence and remission in Italy from 1940 to 2010.

**Methods:**

The subjects were randomly sampled from the general Italian population between 1991 and 2010 in the three population-based multicentre studies: ECRHS, ISAYA, and GEIRD. Individual information on the history of asthma (age at onset, age at the last attack, use of drugs for asthma control, co-presence of hay-fever) was collected on 35,495 subjects aged 20–84 and born between 1925–1989. Temporal changes in rates of asthma incidence and remission in relation to age, birth cohort and calendar period (APC) were modelled using Poisson regression and APC models.

**Results:**

The average yearly rate of asthma incidence was 2.6/1000 (3,297 new cases among 1,263,885 person-years). The incidence rates have been linearly increasing, with a percentage increase of +3.9% (95%CI: 3.1–4.5), from 1940 up to the year 1995, when the rates begun to level off. The stabilization of asthma incidence was mainly due to a decrease in the rates of atopic asthma after 1995, while non-atopic asthma has continued to increase. The overall rate of remission was 43.2/1000person-years, and it did not vary significantly across generations, but was associated with atopy, age at asthma onset and duration of the disease.

**Conclusions:**

After 50 years of a continuous upward trend, the rates of asthma incidence underwent a substantial stabilization in the late 90s. Despite remarkable improvements in the treatment of asthma, the rate of remission did not change significantly in the last seventy years. Some caveats are required in interpreting our results, given that our estimates are based on self-reported events that could be affected by the recall bias.

## Introduction

Many studies have shown that the prevalence of asthma has been rising all over the world since the late 70’s [1]. Recent reports document that it is still increasing in many Westernized countries [2–5]. The causes of this epidemic are still not known. Furthermore, the specific temporal trends in asthma incidence and remission have not been thoroughly investigated, due to the few numbers of studies allowing the incidence to be estimated and their methodological differences.

The study of the temporal variations in asthma incidence may help health-care providers and decision makers to anticipate the burden of asthma and optimize clinical and public health strategies. Furthermore, understanding whether these time trends are related to age, calendar period or birth cohort might offer insights into the aetiology of the disease. In fact, temporal trends may reflect variations in the distribution of the risk factors of asthma. According to some authors, if the increase in environmental determinants is the cause of an epidemic, an increased period effect (indicative of changes in risk factors that affect all ages equally) should be expected. On the other hand, if perinatal exposure to some risk factors is the main cause of the epidemic, a strong cohort effect should be present [6,7].

Finally, changes over time (independent of age) in the rates of remission of asthma may reflect changes in the degree of severity of the disease, in the frequency of asthma under treatment or improvements in available treatment options [8].

In this study we aimed to estimate:

- the effects of age, period and cohort on the incidence of asthma in Italy,
- the temporal variation in the rate of asthma remission during the period from 1940 to 2010.

## Materials and Methods

### Study design

This analysis studied 38,596 subjects, aged 20–84, born between 1925 and 1989, who were recruited between 1991 and 2010 in the three population-based multicentre studies, using the same design: the Italian arm of the European Community Respiratory Health Survey (ECRHS, n = 6,031) [9], the Italian Study on Asthma in Young Adults (ISAYA, n = 18,873) [10], and the Genes Environmental Interaction in Respiratory Diseases (GEIRD) study (n = 13,692) (S1 Text) [11]. In all the studies, a screening questionnaire enquiring about respiratory symptoms, the past and present history of asthma, the presence of hay fever and the use of asthma drugs was sent by post to each subject up to 3 times in the case of non-response. A telephone interview was administered to the remaining non- responders. The questionnaire used was always the same [12], except in the case of the first ECRHS cross-sectional survey.

Ethics approval was obtained in each centre from the appropriate ethics committee. All participants were fully informed about all aspects of the research project and consented to complete and return the questionnaires.

### Asthma incidence analysis and age-period-cohort analysis

#### Asthma Incidence

The presence of lifetime asthma and age at onset of asthma were identified on the basis of a positive answer (at baseline or during the follow-up) to the following questions: *“Have you ever had asthma*?*”*; *“How old were you when you had your first attack of asthma*?*”* [13].

Incidence rates were computed by dividing the number of events (asthma onset) by the total number of person-years at risk. Person-years were defined as the time (in years) a subject was at risk for development of asthma, i.e. the time from birth to the age of the first attack of asthma, if the subject had asthma, or the time from birth until the date of the last interview, if he/she did not have asthma. In the case of follow-up, age at onset of asthma was computed at the time of the first reporting [14]. Atopic asthma was considered present if a subject reported both asthma and allergic rhinitis (i.e. if a subject also replied affirmatively to the question “Do you have any nasal allergies including hay fever?”)

### Age-Period-Cohort (APC) models

Poisson regression models were used to test the effects of age, period and cohort (coded in 5-year intervals) on the incidence of asthma: five models were fitted to data: age; age–drift; age-cohort; age-period; age–period–cohort. In these models, the term *drift* denotes a variable for the overall linear time-trend, which cannot be attributed univocally to cohort or period effects, while the period and cohort parameters are estimates of a non-linear effect [7,15]. The effects of age, period and cohort were tested computing the change in deviance associated to suitable models, according to usual principles of the generalized linear models theory [15]

The assessment of the age-period-cohort effects on asthma incidence were based on the following model:

$$\log\left(\lambda (a,p)\right)=f_{p}(a)+\delta (p)+g(p)+h(c)$$

where λ refers to the asthma rates; *f*, *g*, and *h* are functions; **a**, **p**, and **c** refers to the age, period, and cohort parameters, respectively; the drift parameter “*δ*” is set in the model to deal with the intrinsic non-identifiability problem in APC analysis and to improve estimate interpretation.[7]

To report the three time-parameter effects in a sensible, understandable, and recognizable way, the Carstensen’s restricted cubic spline method was fitted, using 5 knots for age, period and cohort variables [7]. Period effects were calculated as rate ratios, relative to the reference period (1975, which is half-way through the study), while cohort effects were constrained to be null on average with null slope. Age-effects were shown as rates for the reference period. APC models were estimated separately for males and females and for atopic and non-atopic asthma. APC modeling was carried out using the apc.fit function in the Epi package in the R program (version 3.0.2) [7].

### Analysis of asthma remission

The analysis of asthma remission was limited to those subjects who had had asthma during their lives. A subject was considered in remission if he/she had not had an asthma attack in the two years before the most recent interview and replied negatively to the following question: *“Are you currently taking any medicines (including inhalers*, *aerosols or tablets) for asthma*?*”*. The duration of the disease (time since onset) was computed as the time between the first and the last asthma attack for subjects in remission, or as the time between the age at the first asthma attack and the age at the last interview for current asthmatics.

Due to the low number of asthmatic subjects with remission, APC models were not used for this analysis, but survival techniques were applied to the data, using the presence or absence of remission as 'failure', and the time since the onset as *'time at risk'*. A Poisson regression model was used to assess the association of the remission of asthma with the potential prognostic factors (sex, age at onset, presence of hay-fever, birth cohort). The statistical analysis was performed using STATA software, release 13.1.

### Sensitivity Analyses

Three sensitivity analyses were performed to estimate the size of the potential bias due to retrospective approach and to potential differential recall on the incidence and remission of disease [16]. Details are included in the supporting information (S2 Text).

## Results

### Participants and response rates

In total, 38,596 out of 57,769 eligible individuals (average response rate: 67%) took part in one of the three surveys (ECRHS, ISAYA or GEIRD) and answered at least one of the relative screening questionnaires. Of these, 2,144 subjects were excluded because they had only participated in the 1991–93 screening, which did not include the question about age at onset, and further 987 subjects were excluded because they had some missing data on the main variables. The analyses were carried on the remaining 35,495 subjects with complete information.

### Rates of asthma incidence

Overall, 3,297 incident cases of asthma occurred among 1,263,885 person-years (py) (Table 1). The average crude rate of asthma incidence was 2.6/1,000py. The subjects of the ECRHS survey had lower rates of asthma incidence than those of the ISAYA and GEIRD surveys. The rate of asthma incidence was 7-fold greater in subjects with allergic rhinitis than in subjects without.

**Table 1:** Person-years, number of subjects reporting ever having had asthma, and the crude incidence (95%CI) of asthma by sex, age at onset, birth cohort, and period, type of study and presence of hay fever.

	Person-years (py)	No. of cases of asthma	Incidence rates/1,000 py (95%CI)
**Sex**			
women	646,577	1,618	2.5 (2.4–2.6)
men	617,309	1,679	2.7 (2.6–2.9)
**Age at onset (y)**			
0–10	345,653	1,692	4.9 (4.7–5.1)
10–20	335,050	693	2.1 (1.9–2.2)
20–30	294,244	465	1.6 (1.4–1.7)
30–40	184,936	320	1.7 (1.6–1.9)
40–50	66,890	97	1.5 (1.2–1.8)
50–60	23,516	21	0.9 (0.6–1.4)
>60	13,596	9	0.7 (0.3–1.3)
**Birth Cohort**			
1925–39	45,940	40	0.9 (0.6–1.2)
1940–9	85,907	99	1.2 (0.9–1.4)
1950–9	306,492	490	1.6 (1.5–1.7)
1960–9	493,129	1,272	2.6 (2.4–2.7)
1970–9	283,028	1,125	4 (3.7–4.2)
1980–9	49,390	271	5.5 (4.9–6.2)
**Period**			
1925–39	4,106	2	0.5 (0.1–2.0)
1940–9	12,174	10	0.8 (0.4–1.5)
1950–9	50,485	92	1.8 (1.5–2.2)
1960–9	160,216	470	2.9 (2.7–3.2)
1970–9	283,859	882	3.1 (2.9–3.3)
1980–9	328,020	899	2.7 (2.6–2.9)
1990–9	305,755	673	2.2 (2.0–2.4)
2000–9	119,954	269	2.2 (2.0–2.5)
**Survey**			
ECRHS	160,741	309	1.9 (1.7–2.1)
ISAYA	601,480	1,595	2.7 (2.5–2.8)
GEIRD	501,665	1,393	2.8 (2.6–2.9)
*GEIRD – 20–44yrs*	*324*,*471*	*1*,*161*	*3*.*6 (3*.*4–3*.*7)*
*GEIRD – 45–64yrs*	*108*,*573*	*163*	*1*.*5 (1*.*3–1*.*8)*
*GEIRD – 65–84yrs*	*68*,*621*	*69*	*1*.*0 (0*.*8–1*.*3)*
**Hay fever**			
No	1,016,831	1,248	1.2 (1.2–1.3)
Yes	240,365	2,023	8.4 (8.1–8.8)
**TOTAL**	**1,263,885**	**3,297**	**2.6 (2.5–2.7)**

**Fig 1:**
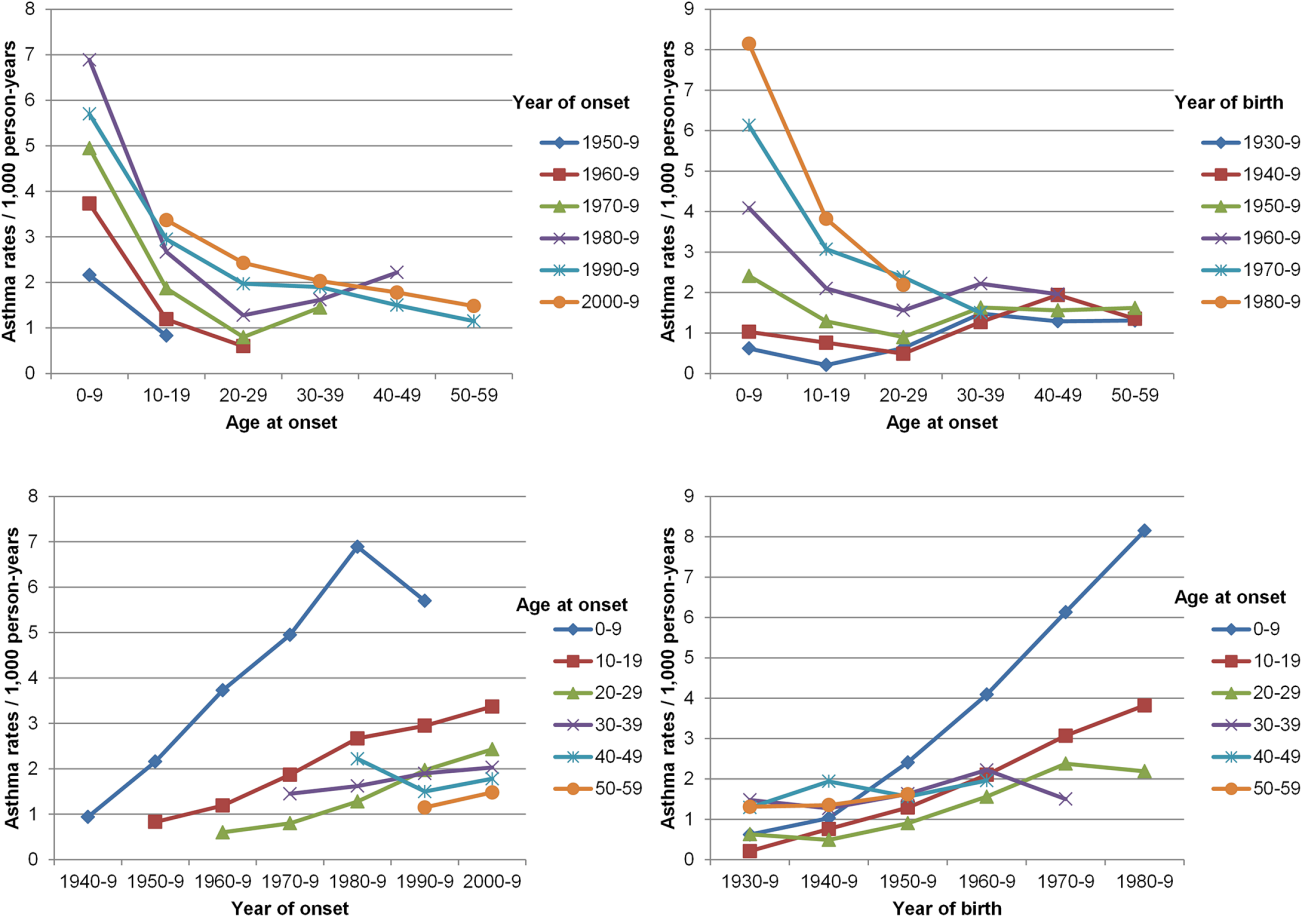
Age-specific rates of asthma in Italy by period of asthma onset and birth cohort: **A)** Age-specific rates by year of onset: age at onset on x axis, with rates corresponding to same period connected by lines; **B)** Age-specific rates by birth cohort: age at onset on x axis, with rates corresponding to same cohorts connected by lines; **C)** Period-specific rates by age: year of onset on x axis, with rates corresponding to same age groups connected by lines; **D)** Cohort-specific rates by age: year of birth on x axis, with rates corresponding to same age groups connected by lines.

Fig 1 summarizes the crude age-specific rates of asthma incidence, stratified by period of asthma onset and by birth cohort. Children in the 0–9yrs age-group had the highest rates, which steeply decreased until the age of 20–29yrs. For all the age-groups, the incidence rates of asthma increased in recent years, and this trend was particularly evident in the case of younger subjects. The age-specific rates of asthma were higher in more recent birth cohorts and periods (person years, events and crude rates by age and cohort are reported in S1 Table).

### Age-Period-Cohort “effects” on asthma incidence

**Fig 2:**
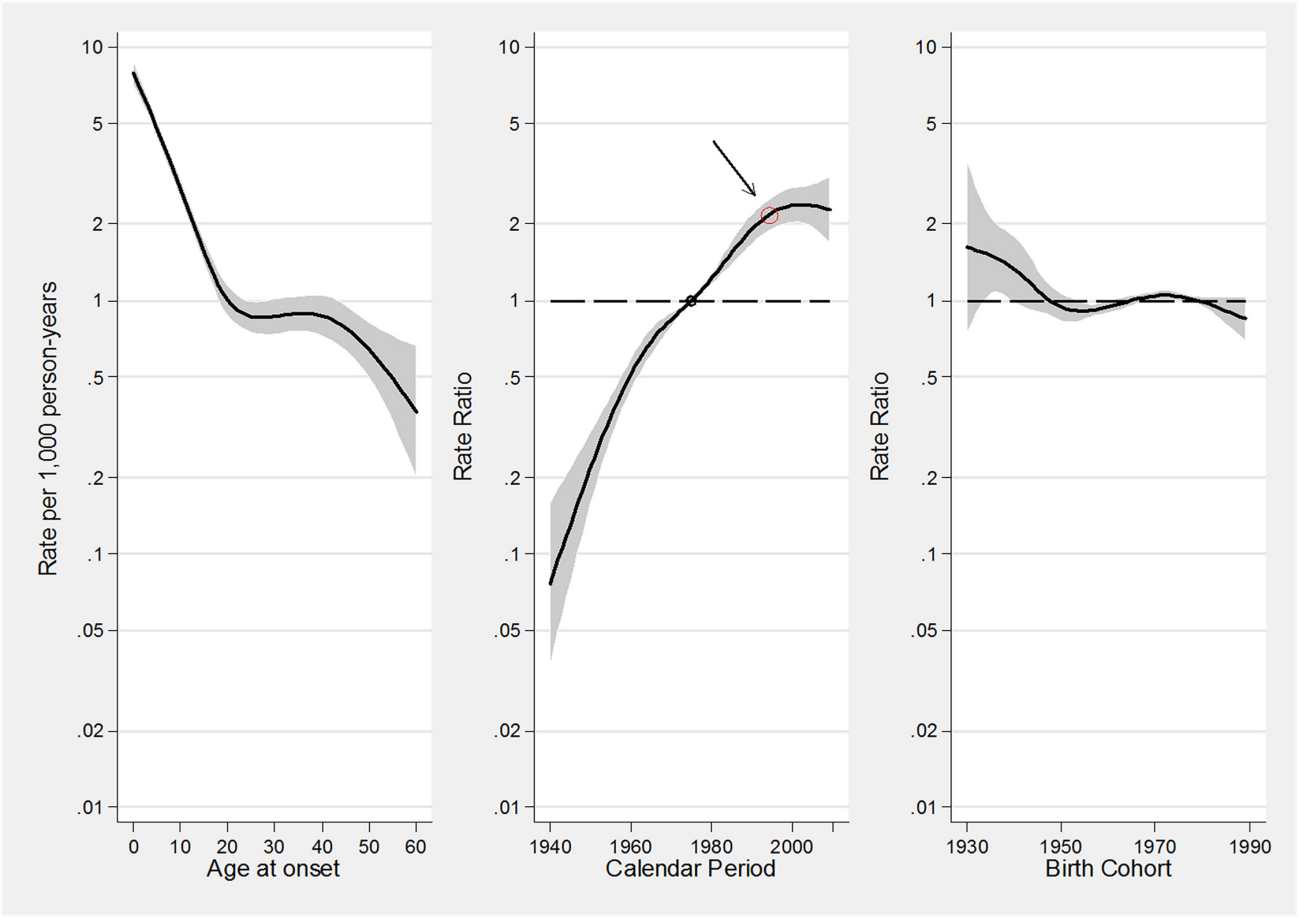
A-C. Estimated effects from the APC model for asthma incidence rates. Fig 2A represents the age-specific incidence rates, referred to the reference period 1975; Fig 2B represents the merged period and drift effects (rate ratios): the linear increasing trend is given by the drift, while deviations from linearity (curvature) are determined by the period effect; Fig 2C represents the birth cohort effect. The arrow in B indicates the calendar period when the incidence of asthma has begun to level off. The respective regions surrounding the lines provide the 95% confidence intervals.

The results of fitting Poisson regression models to the incidence data (Table 2), suggested that the linear time trend (“*drift”*) was the most relevant determinant to explain the variation over time of asthma incidence rates. Moreover, a statistically significant component related to period emerged while there was no statistically significant effect due to the cohort (Fig 2).

**Table 2:** Comparison of age-period color sub-models for asthma rates in males and females and for atopic and non-atopic asthma in Italy.

Model	Residual deviance (residual df)	Effect	Change in deviance (df)	p-value
**OVERALL**				
Age	403.63 (158)	A		
Age-drift(cohort)	217.32 (157)	Δ†|A	186.31 (1)	<0.0001
Age-Cohort	211.76 (153)	C‡|A	5.56 (4)	0.235
Age-Period-Cohort	198.24 (149)	P‡|A,C	13.53 (4)	0.009
Age-Period	201.09 (153)	C‡|A,P	-2.85 (-4)	0.583
Age-drift(period)	217.32 (157)	P‡|A	-16.23 (-4)	0.003
**Males**				
Age	408.05 (158)	A		
Age-drift(cohort)	220.04 (157)	Δ†|A	188.01 (1)	<0.0001
Age-Cohort	214 (153)	C‡|A	6.04 (4)	0.196
Age-Period-Cohort	200.69 (149)	P‡|A,C	13.31 (4)	0.010
Age-Period	203.47 (153)	C‡|A,P	-2.78 (-4)	0.595
Age-drift(period)	220.04 (157)	P‡|A	-16.57 (-4)	0.002
**Females**				
Age	385.2 (158)	A		
Age-drift(cohort)	218.42 (157)	Δ†|A	166.78 (1)	<0.0001
Age-Cohort	213.18 (153)	C‡|A	5.25 (4)	0.263
Age-Period-Cohort	203.42 (149)	P‡|A,C	9.76 (4)	0.045
Age-Period	208.2 (153)	C‡|A,P	-4.78 (-4)	0.310
Age-drift(period)	218.42 (157)	P‡|A	-10.22 (-4)	0.037
**Atopic asthma**				
Age	540.85 (182)	A		
Age-drift(cohort)	287.02 (181)	Δ†|A	253.832 (1)	<0.0001
Age-Cohort	281.29 (177)	C‡|A	5.728 (4)	0.220
Age-Period-Cohort	242.88 (173)	P‡|A,C	38.411 (4)	<0.0001
Age-Period	246.62 (177)	C‡|A,P	-3.736 (-4)	0.443
Age-drift(period)	287.02 (181)	P‡|A	-40.403 (-4)	<0.0001
**Non-atopic asthma**				
Age	337.09 (182)	A		
Age-drift(cohort)	221.9 (181)	Δ†|A	115.195 (1)	<0.0001
Age-Cohort	213.96 (177)	C‡|A	7.935 (4)	0.093
Age-Period-Cohort	211.91 (173)	P‡|A,C	2.05 (4)	0.727
Age-Period	221.05 (177)	C‡|A,P	-9.141 (-4)	0.058
Age-drift(period)	221.9 (181)	P‡|A	-0.845 (-4)	0.932

Age, period and birth cohorts are modelled in 5-years periods. A: age effect; P: period effect; C: cohort effect

† drift (linear) estimate

‡ curvature estimate

**Fig 3:**
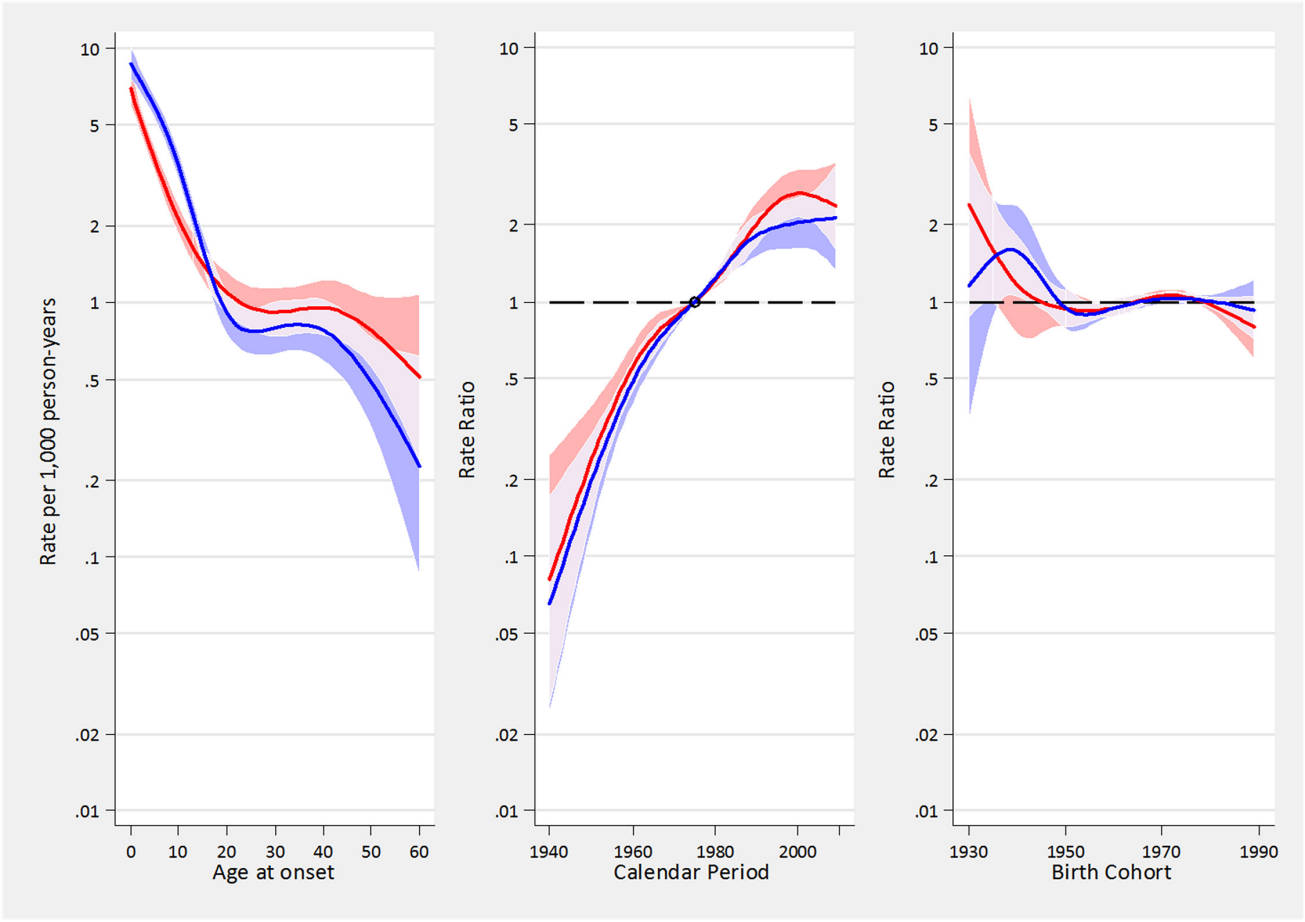
A-C. Estimated effects from the APC models for asthma incidence rates in males (blue tonalities) and females (red tonalities). Fig 3A represents the age-specific incidence rates, referred to the reference period 1975; Fig 3B represents the merged period and drift effects (rate ratios), with linear increasing trend determined by the drift, and deviations from linearity (curvature) determined by the period effect; Fig 3C represents the birth cohort effect. The respective regions surrounding the lines provide the 95% confidence intervals (95%CI). Overlapping 95%CI are highlighted with lighter areas.

APC effects were similar in men and women. Fig 3 shows the effects of age, period and cohort on asthma incidence in males and females. The rates of asthma constantly increased from 1940 up to 1995/2000, when the rates underwent a substantial stabilization, interrupting the linear increase. Overall, the age-adjusted relative annual increase in asthma incidence rates during the period 1940–2010 was +3.9% (95%CI: 3.1–4.5), and this percentage increase was almost identical in males (+4.0%, 95%CI: 3.3–4.7) and in females (+3.9%, 95%CI: 3.4–4.3). Taken as a whole, the asthma incidence had a 10-fold increase in the last 70 years, and it doubled between 1980 and 2000 (S3 Text).

**Fig 4:**
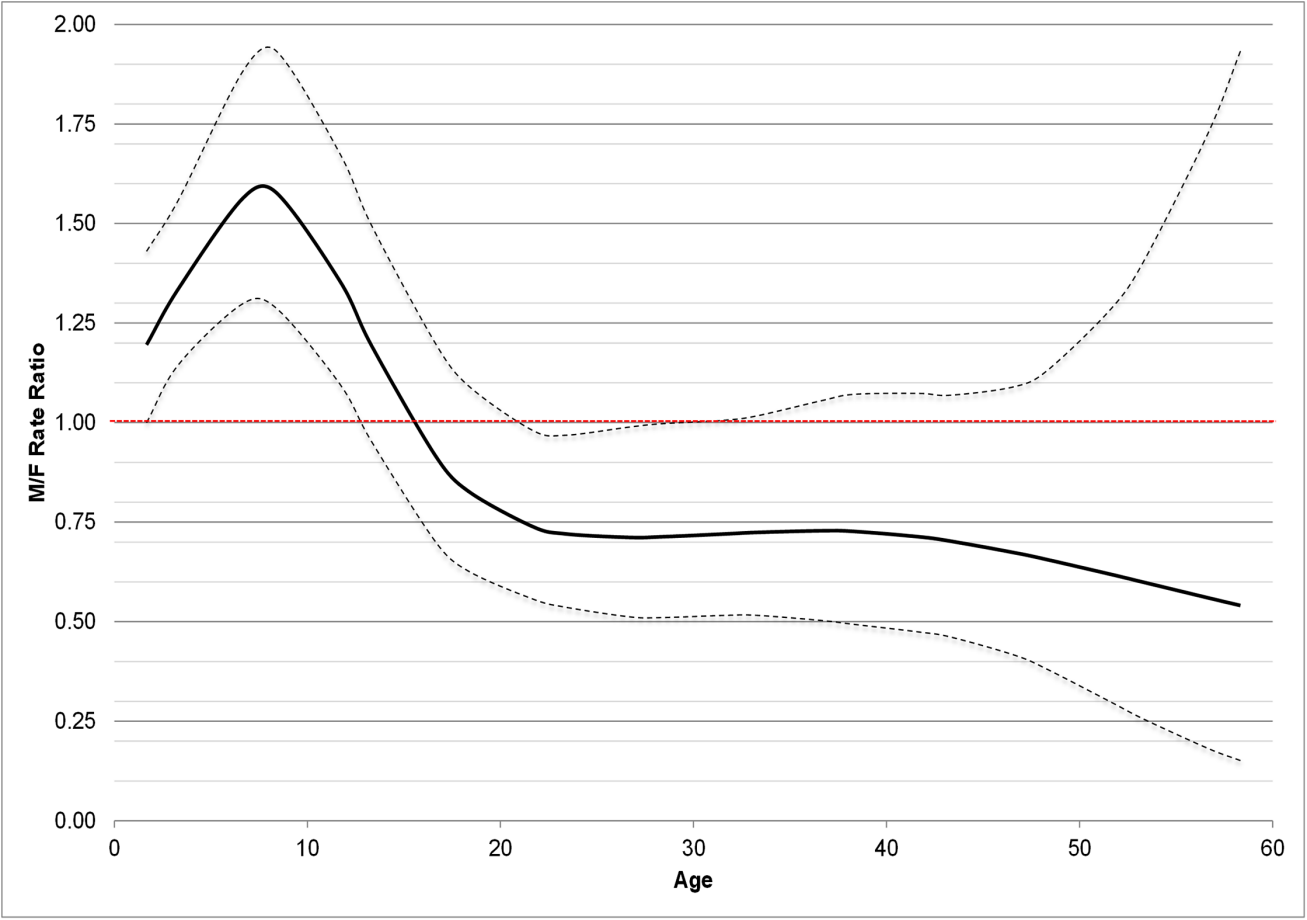
Incidence rate ratios (RR, with 95%CI) of asthma by age in men vs. women.

In both genders, the rates of asthma incidence exponentially decreased from birth to the age of 25, were almost stable from 25 to 45, and then decreased again after the age of 45. During childhood, the rates of asthma incidence were higher in boys than girls, they were similar around puberty and then they became 30–50% lower in men than in women after the age of 20. (Fig 4)

**Fig 5:**
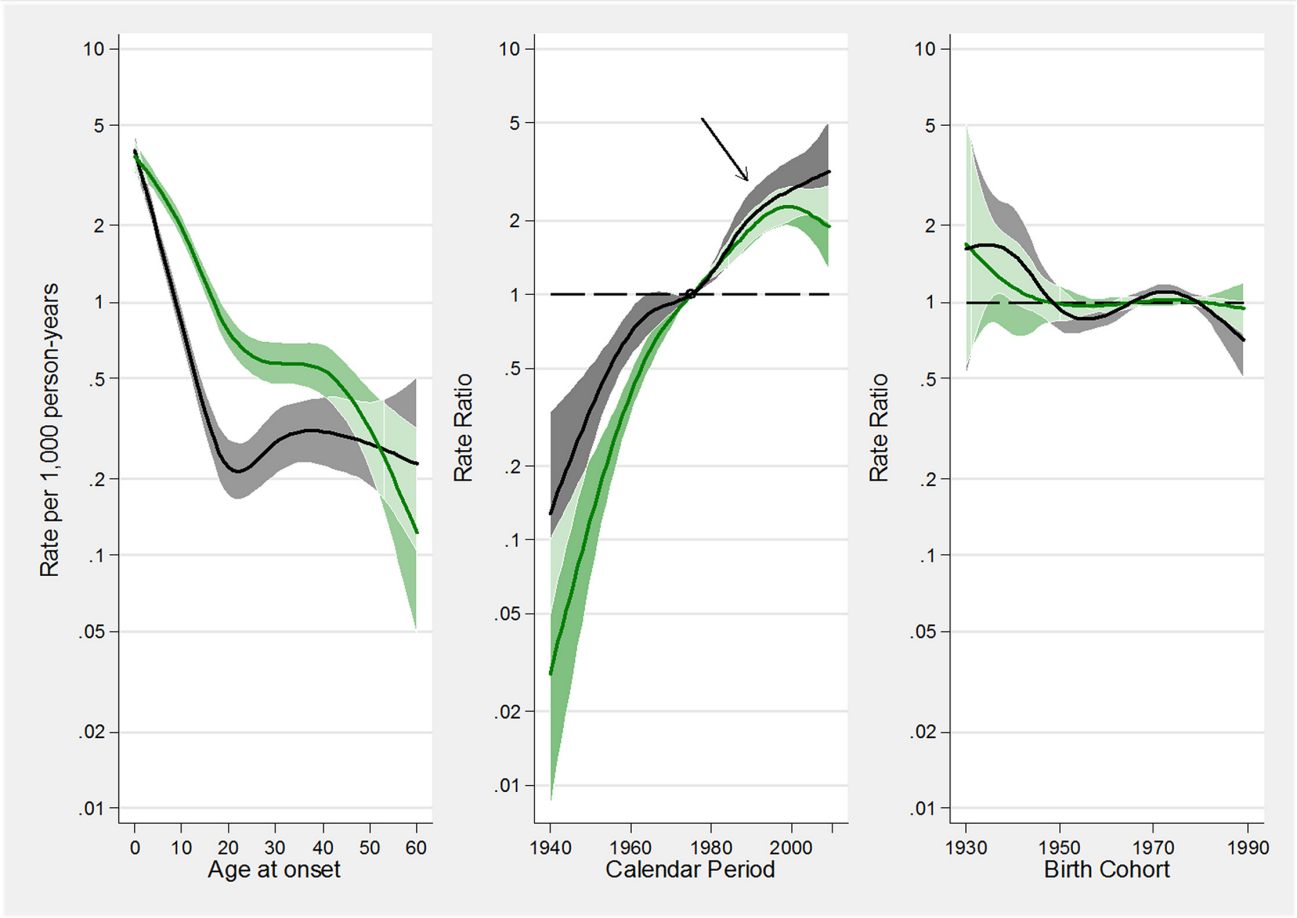
A-C. Estimated effects from the APC models for incidence rates of atopic asthma (green tonalities) and non-atopic asthma (grey tonalities). Fig 5A represents the age-specific incidence rates, referred to the reference period 1975; Fig 5B represents the merged period and drift effects (rate ratios), with linear increasing trend determined by the drift, and deviations from linearity (curvature) determined by the period effect; Fig 5C represents the birth cohort effect. The arrow in B indicates the calendar period when the incidence of asthma has begun to level off. The respective regions surrounding the lines provide the 95% confidence intervals (95%CI). Overlapping 95%CI are highlighted with lighter areas.

The leveling-off of the continuous linear increase in asthma incidence, occurring in 1995/2000, was mainly caused by the decrease in the incidence of asthma in allergic subjects (Table 2 and Fig 5). Indeed there was a strong and statistically significant period effect in allergic but not in non-allergic subjects, meaning that the incidence of asthma no longer increased after 1995/2000 in allergic subjects, while it continued its linear upward trend in non-allergic subjects.

### Asthma remission

212 subjects with asthma were excluded from the remission analysis because of missing or incongruent data at the questions on use of drugs for asthma or age at the last asthma attack. Fifty-two percent of asthmatic subjects (1,610 out of 3,087) remitted. Subjects in remission had an earlier mean age at onset (9.9yrs vs.19.4yrs, p<0.001) and a shorter duration of the disease than subjects with current asthma (6.9yrs vs.17.7yrs, p<0.001). Overall, the rate of remission was 43.2/1000py. The rates of remission were higher in men than in women, in more recent generations, and in subjects without hay fever (Table 3). Furthermore, the rates of remission were higher in people who developed asthma in childhood than in adulthood, and peaked in the first years after the onset. When adjusting for all potential confounders, the variables that were statistically associated with the rate of remission were the presence of hay fever, the age at onset and the time since onset of asthma. The rate of remission was stable across birth cohorts.

**Table 3:** Number of subjects with asthma and asthma remissions, crude incidence and risk ratios (RR) of asthma remission adjusted by sex, presence of hay fever, age at onset, birth cohort, and time since onset.

	Subjects	Remissions	Time at risk	Remission rates	Risk ratios
	n	n (%)	Person-years	per 1000 person-years (95% CI)	adjusted RR (95%CI)
**Sex**					
Men	1590	898 (56.5)	19778	45.4 (42.5–48.5)	1 (-)
Women	1497	712 (47.6)	17454	40.8 (37.9–43.9)	0.94 (0.85–1.04)
**Hay fever**					
No	1165	769 (66.0)	10843	70.9 (66.1–76.1)	1 (-)
Yes	1898	825 (43.5)	26118	31.6 (29.5–33.8)	0.48 (0.43–0.53)
**Birth cohort**					
before 1959	566	275 (48.6)	8400	32.7 (29.1–36.8)	1 (-)
1960–1969	1183	626 (52.9)	14905	42.0 (38.8–45.4)	1.08 (0.93–1.25)
after 1970	1338	709 (53.0)	13926	50.9 (47.3–54.8)	1.07 (0.92–1.25)
**Age at onset (years)**					
0–14	1927	1256 (65.2)	26901	46.7 (44.2–49.3)	1 (-)
15–29	750	270 (36.0)	7512	35.9 (31.9–40.5)	0.73 (0.42–0.67)
30+	410	84 (20.5)	2820	29.8 (24.1–36.9)	0.53 (0.41–0.53)
**Time since the onset (years)**					
0–9	3087	1255 (41.7)	20749	60.5 (57.2–63.9)	1 (-)
10–19	1,358	272 (20.0)	9992	27.2 (24.2–30.7)	0.47 (0.19–0.31)
20–29	678	65 (9.6)	4502	14.4 (11.3–18.4)	0.24 (0.19–0.31)
30+	266	18 (6.8)	1989	9.0 (5.7–14.3)	0.15 (0.09–0.21)
**TOTAL**	**3087**	**1610**	**37232**	**43.2 (41.2–45.4)**	

CI: confidence interval

### Sensitivity analyses

The sensitivity analyses (reported in the online repository) confirmed the main results, suggesting that the recall bias and the retrospective approach might have affected our results only by a minor extent.(S2 Text)

## Discussion

The main results of the analyses on incidence and remission of asthma are:

- Asthma incidence steadily and continuously increased from 1940 to 1995, both in men and women, with an average relative increase of 3.9% each year. This temporal trend cannot be univocally attributed to period or cohort effects.
- Since 1995/2000 the incidence of asthma has levelled off. This “late period effect” was mainly caused by the decrease of the incidence of *atopic* asthma, while the incidence of *non-atopic* asthma is continuously increasing.
- After adjusting for period and cohort effects, the incidence of asthma showed a non-linear, downward trend with ageing.
- The likelihood of asthma remission did not significantly vary across generations, despite improvements in the treatment of the disease.

### Trends in incidence of asthma over time

It has been suggested that the still increasing trend in the prevalence of asthma reported in many countries might be attributable to an increase in the incidence of the disease [1–5]. However, little is known about the real temporal trend in the onset of asthma. The few surveys that have estimated the incidence of asthma in different populations have shown that incidence has tended to be higher in later studies, suggesting a rise in asthma incidence [3,17,18].

This is one of the few studies that allowed the direct assessment of temporal trends in asthma incidence in the last seventy years, thanks to the availability of different birth cohorts from the same general Italian population, where the history of asthma was assessed at different times with the same standardized methods. Our results document that the incidence of both atopic and non-atopic asthma has increased almost linearly from 1940 to 1995/2000, with a relative annual increment of 3.9%. In the same period, a continuous increase in allergic sensitization has been reported [19,20].

In our analysis, APC-models, which included both the *drift* (i.e., the linear variation of the incidence in time that is not univocally attributable to a calendar-period or a cohort effect) and the age, the period, and the cohort effects, were fitted to the data [7]. During the period 1940–1995, the *“drift*” was the only statistically significant component. Accordingly, it was impossible to ascribe this increase to either a specific cohort or a calendar period effect. This suggests that the rising incidence over time was due to an increased susceptibility that has affected all age groups and generations since 1940. This pattern might reflect either increased population exposures to widespread environmental determinants (allergens, pollutants, occupational exposures) or lifestyles, such as those associated with increased levels of urbanization. At least to a certain extent, this might also reflect an increased public and professional awareness of asthma symptoms that has led to a larger diagnostic labeling of asthma in more recent decades. The fact that the rising trend in incidence was the same for men and women documents that it is not caused by gender-specific lifestyles or exposures.

The leveling off in asthma incidence after 1995/2000 seems to be driven by a “late period effect” linked to a stabilization of incidence of atopic asthma, which represents the majority of asthma cases, while the incidence of non-atopic asthma has continued to increase. This finding agrees with a recent paper, which showed that non-atopic asthma was responsible for the increase of asthma prevalence registered in the last decade in Italy [2].

The stabilization, or slight decrease, of the incidence of atopic asthma in the last ten years might be due to a reduction in environmental exposures or to more effective “allergen avoidance” strategies. However a potential alternative explanation could be that the “ceiling” level of atopic asthma prevalence has been attained, meaning that the maximum effect of changing environmental exposure in susceptible individuals has been already seen, and the proportion of the population that has the potential of acquiring asthma has been reached [21].

It’s worth noting that non-atopic asthma has also been linked to smoking habits. However, the incidence of non-atopic asthma is still increasing in Italy in spite of a decreasing trend in smoking habits [22].

### Incidence of asthma by sex and age

Our results in the age-sex-related pattern of incidence over the last seventy years, confirmed what was observed in previous Italian and US studies using shorter calendar-time windows [23,24]. Children, especially in an early age, experienced greater rates of incident asthma than adults. Boys had higher rates of asthma than girls until puberty, while the reverse occurred in adulthood. Moreover, our findings indicate that women continued to have a greater incidence of asthma, even in their forties and fifties, at the onset of hormonal changes that characterize menopause.

Our study also investigated the incidence of asthma among older subjects. In agreement with Winer [ 24 ], we found that the incidence rates in both sexes decreased after the age of 40. This downward trend parallels the decrease of allergic sensitization in older subjects and replicates the natural history of other atopic diseases, such allergic rhinitis and eczema [25]. It is worthwhile to mention that the decrease of asthma incidence after the age of 40 may partly reflect the doctors’ differential propensity to diagnose asthma or COPD according to the age of their patients [26]. Indeed, distinguishing between asthma and COPD can be quite challenging, even for the most expert physician, and COPD is often misdiagnosed as asthma in young people, while the opposite happens in the elderly [27].

### Asthma remission

Despite the decreasing trend of incidence, the prevalence of asthma is still rising in Italy and in many other countries [2,28]. This is because prevalence is a balance between incidence and remission. The latter has to increase more quickly than the former in order to significantly reverse the increasing trend of asthma in the last decades.

The average yearly rate of remission in Italy during the last seventy years was 43.2 per 1000 patients and did not significantly change over time (birth-cohorts). Remarkable improvements in the treatment of asthma occurred in the same period: inhaled corticosteroids (ICS) were introduced in the early 1970s and they became the first line therapy for patients with asthma by the late 1980s [29].

Accordingly, our results document that despite these advances in therapy, which have contributed to decline in asthma mortality [30], the remission of asthma has not significantly improved in the last decades. This finding reflects either the fact that, up to now, asthma cannot be cured but only controlled [31], and that patient variability in the severity and course of the disease and poor patient adherence to therapy are historical barriers to reduce the burden of asthma. Remission in asthma mainly occurs in patients with a mild form of the disease and it is rarely observed among more severe subjects with poor lung function or a high level of symptoms at baseline [32].

Furthermore, our results suggest that the baseline clinical characteristics of the patients are the main determinants of prognosis of asthma. The rates of asthma remission were higher in subjects who had developed asthma early in childhood and in those with a shorter duration of the disease. The presence of allergic rhinitis, which was used as proxy of atopy, was a strong marker of the persistence of asthma. These results are in agreement with previous studies, which separately reported the rates of asthma remission in children and in adults [33–35].

### Strengths and Limitations

This study used the data collected in three large, epidemiological, postal-screening surveys carried out in Italy between 1991 and 2010. The response rate was quite high overall compared to other similar multicenter studies on adults. The large number of person-years considered (nearly 1.3million), and the variety of ages, periods and cohorts considered in the analyses are the main strengths of this study.

The Italian centers involved in the study were not chosen randomly but on the basis of the availability of research teams that were able to carry out the survey. However, the study areas are spread all over Italy and well represent the geographical (North versus South, Sub-Continental versus Mediterranean) features of the country. Furthermore, in each center, the subjects included in this study were randomly selected from the general population. The prevalence of asthma obtained from the GEIRD study [2] is very similar to that obtained in a representative national survey on adults, performed in the same period, using information from the Health Search Database owned by the Italian College of General Practitioners [36], suggesting that the results obtained in our sample are likely to be representative of the general population of Italian adults. One limitation is that the history of asthma was self-reported by the subjects, and not confirmed by a clinical evaluation. However, the validity of the questions used in the surveys had been tested in previous studies, showing good sensitivity and specificity [13,37], and this definition has been adopted in several international studies [38].

Moreover, the information collected relies on the recall of the subjects. The estimates of asthma incidence and remission are therefore potentially underestimated particularly in older subjects, because elderly people who had an early onset and remission of the disease may more frequently forget that they had asthma, than younger people with a more reliable recall. As a consequence, this differential recall bias could have distorted the estimates of the time trends and the assessment of the determinants of asthma incidence and remission. However, even if we cannot completely exclude the presence of a possible recall bias, the sensitivity analyses performed to assess the extent of the bias due to a possible differential recall in young and elderly people suggest that the recall bias might have influenced the results of the main analyses only to a minor extent. One more limitation is that atopy was not clinically assessed, but the presence of allergic rhinitis was considered a proxy of atopy in the subjects with asthma. However, a recent publication by our group reported that 87% of subjects with both asthma and allergic rhinitis had positive skin prick tests, while only 33% of subjects with asthma but not allergic rhinitis had positive skin prick tests [39], indicating a discrete validity of the question on allergic rhinitis in distinguishing allergic from non-allergic asthma.

### Conclusions

After 50 years of a continuous upward trend, the rates of asthma incidence underwent a substantial stabilization in the mid-90s. The leveling off of the incidence was mainly due to the decrease of the incidence of atopic asthma, while non-atopic asthma incidence has continued to increase. Despite remarkable improvements in the treatment of asthma, the rate of remission has not significantly changed in the last seventy years. Although the validity and the reliability of the self reported history of asthma have been proved good, the recall bias might have influenced our results to some extent. This potential source of error has to be taken into account when interpreting our findings.

## Supporting Information

S1 Table Number of incident cases of asthma, person-years at risk, and incidence rates of asthma (per 1,000 person-years) by age at onset and calendar period, modeled in 10-years time intervals. (DOCX)

S1 Text Surveys included in the analyses. (DOCX)

S2 Text Sensitivity analyses. (DOCX)

S3 Text Estimates of age-period specific rates of asthma between 1940–2010. (DOCX)
