# Correction: Seventy Years of Asthma in Italy: Age, Period and Cohort Effects on Incidence and Remission of Self-Reported Asthma from 1940 to 2010

**DOI:** 10.1371/journal.pone.0191589

**Published:** 2018-01-17

**Authors:** Giancarlo Pesce, Francesca Locatelli, Isa Cerveri, Massimiliano Bugiani, Pietro Pirina, Ane Johannessen, Simone Accordini, Maria Elisabetta Zanolin, Giuseppe Verlato, Roberto de Marco

The following information is missing from the Funding section: This study was supported by the Italian "Ministry of Health" (Ministero della Salute) via CUP number H91J11000220001.
