# Supplementary material for: Seventy Years of Asthma in Italy: Age, Period and Cohort Effects on Incidence and Remission of Self-Reported Asthma from 1940 to 2010
Source: PLoS One. 2015 Oct 6;10(10):e0138570. doi: 10.1371/journal.pone.0138570 (PMC4595078; doi:10.1371/journal.pone.0138570)
Supplement: S1 Table — (DOCX) [file pone.0138570.s001.docx]

**S1 Table. Number of incident cases of asthma, person-years at risk, and incidence rates of asthma (per 1,000 person-years) by age at onset and calendar period, modeled in 10-years time intervals.**

|  | **CALENDAR PERIOD** | | | | | | | |  |
| --- | --- | --- | --- | --- | --- | --- | --- | --- | --- |
| **AGE** | <1940 | 1940-9 | 1950-9 | 1960-9 | 1970-9 | 1980-9 | 1990-9 | 2000-9 | **Total** |
|  |  |  |  |  |  |  |  |  |  |
| *Cases* | | | | | | | | | |
| 0-9 | 1 | 8 | 83 | 414 | 631 | 353 | 39 |  | **1,529** |
| 10-9 | 1 | 0 | 7 | 45 | 202 | 327 | 144 | 22 | **748** |
| 20-9 |  | 2 | 2 | 5 | 30 | 136 | 233 | 68 | **476** |
| 30-9 |  |  | 0 | 5 | 12 | 60 | 194 | 94 | **365** |
| 40-9 |  |  |  | 1 | 6 | 18 | 52 | 52 | **129** |
| 50-9 |  |  |  |  | 1 | 5 | 9 | 21 | **36** |
| 60-9 |  |  |  |  |  | 0 | 2 | 8 | **10** |
| 70-9 |  |  |  |  |  |  | 0 | 4 | **4** |
| >80 |  |  |  |  |  |  |  | 0 | **0** |
| **Total** | **2** | **10** | **92** | **470** | **882** | **899** | **673** | **269** | **3,297** |
|  |  |  |  |  |  |  |  |  |  |
| *Person-years* | | | | | | | | | |
| 0-9 | 3,728 | 8,473 | 38,484 | 110,852 | 127,500 | 51,265 | 6,843 |  | **347,145** |
| 10-9 | 380 | 3,334 | 8,405 | 37,816 | 107,869 | 122,634 | 48,826 | 6,535 | **335,799** |
| 20-9 |  | 376 | 3,308 | 8,334 | 37,446 | 106,276 | 118,365 | 27,953 | **302,058** |
| 30-9 |  |  | 374 | 3,288 | 8,282 | 37,013 | 102,084 | 46,249 | **197,290** |
| 40-9 |  |  |  | 371 | 3,229 | 8,123 | 34,734 | 29,240 | **75,697** |
| 50-9 |  |  |  |  | 368 | 3,193 | 7,844 | 14,236 | **25,641** |
| 60-9 |  |  |  |  |  | 367 | 3,182 | 6,701 | **10,250** |
| 70-9 |  |  |  |  |  |  | 367 | 3,156 | **3,523** |
| >80 |  |  |  |  |  |  |  | 363 | **363** |
| **Total** | **4,108** | **12,183** | **50,571** | **160,661** | **284,694** | **328,871** | **322,245** | **134,433** | **1,297,766** |
|  | | | | | | | | | |
| *Incidence rates (per 1,000 person-years)* | | | | | | | | | |
| 0-9 | 0.30 | 0.94 | 2.16 | 3.73 | 4.95 | 6.89 | 5.70 |  | **4.40** |
| 10-9 | 2.63 | 0.00 | 0.83 | 1.19 | 1.87 | 2.67 | 2.95 | 3.37 | **2.23** |
| 20-9 |  | 5.32 | 0.60 | 0.60 | 0.80 | 1.28 | 1.97 | 2.43 | **1.58** |
| 30-9 |  |  | 0.00 | 1.52 | 1.45 | 1.62 | 1.90 | 2.03 | **1.85** |
| 40-9 |  |  |  | 2.70 | 1.86 | 2.22 | 1.50 | 1.78 | **1.70** |
| 50-9 |  |  |  |  | 2.72 | 1.57 | 1.15 | 1.48 | **1.40** |
| 60-9 |  |  |  |  |  | 0.00 | 0.63 | 1.19 | **0.98** |
| 70-9 |  |  |  |  |  |  | 0.00 | 1.27 | **1.14** |
| >80 |  |  |  |  |  |  |  | 0.00 | **0.00** |
| **Total** | **0.54** | **0.82** | **1.82** | **2.93** | **3.10** | **2.73** | **2.09** | **2.00** | **2.54** |
