# Supplementary material for: Seventy Years of Asthma in Italy: Age, Period and Cohort Effects on Incidence and Remission of Self-Reported Asthma from 1940 to 2010
Source: PLoS One. 2015 Oct 6;10(10):e0138570. doi: 10.1371/journal.pone.0138570 (PMC4595078; doi:10.1371/journal.pone.0138570)
Supplement: S1 Text — (DOCX) [file pone.0138570.s002.docx]

**S1 Text. Surveys included in the analyses.**

- the *Italian arm of the European Community Respiratory Health Survey (ECRHS*)^1^ started between 1991 and 1993 (de Marco 1994). Random sample of the general population aged 20 to 44 years (M:F = 1:1) were randomly selected from the general population in each of three Italian centres (3,000 in Turin and Verona, 1,000 in Pavia). 6,031 subjects (response rate=86%) took part to the study in 1991/93, and have been invited to participate to a follow-up in 1999 and 2000 (3,856 responders; response rate=74%). A second follow-up was performed in the centre of Verona (1,420 responders, response rate: 83%) in 2008.
- the *Italian Study on Asthma in Young Adults (ISAYA)*^2^ was carried out on a random sample of about 3000 subjects aged 20 to 44 (M:F = 1:1) from the general population in each of nine Italian centres: Ferrara, Pavia, Pisa, Sassari, Sassuolo, Syracuse, Turin, Udine and Verona. 18,873 subjects were recruited between 1998 and 2000 (response rate=73%). In two centres (Verona and Sassari), a follow-up was performed in 2008/2009 (2,272 responders, response rate: 54%)
- The *Genes Environmental Interaction in Respiratory Diseases (GEIRD)*^3^  study, was conducted between 2007 and 2011 on random samples of about 3000 subjects aged 20– 44 years (male : female=1:1) in each of seven Italian centres: Ancona, Pavia, Terni, Salerno, Sassari, Turin and Verona. Additional random samples of about 1000 subjects aged 45–64 and 65–84 years were selected in four (Turin, Pavia, Verona and Sassari ) and in two centres (Verona and Sassari) respectively. Overall, 13,692 subjects were recruited in GEIRD (response rate: 58%).

**Figure A. Number (with response rate in %) of participating subjects to the screening questionnaire by center in the surveys carried out in Italy between 1991 and 2010.** ECRHS: European Community Respiratory Health Survey; ISAYA: Italian Study on Asthma in Young Adults; GEIRD: Gene-Environment Interactions in Respiratory Diseases. PV: Pavia; TO: Turin; VR: Verona; SS: Sassari; UD: Udine; Sas: Sassuolo; FE: Ferrara; PI: Pisa; SR: Syracuse; AN: Ancona; TR: Terni; SA: Salerno. Arrows indicate follow-ups on the same sample of subjects.

******

**References**

1. Burney PG, Luczynska C, Chinn S, Jarvis D. The European Community Respiratory Health Survey. Eur Respir J 1994;7:954-60.
2. De Marco R, Poli A, Ferrari M, et al. Italian Study on Asthma in Young Adults. Clin Exp Allergy 2002;32:1405-12.
3. De Marco R, Accordini S, Antonicelli L, et al. The Gene-Environment Interactions in Respiratory Diseases (GEIRD) Project. Int Arch Allergy Immunol 2010;152:255-63.
