# Supplementary material for: Seventy Years of Asthma in Italy: Age, Period and Cohort Effects on Incidence and Remission of Self-Reported Asthma from 1940 to 2010
Source: PLoS One. 2015 Oct 6;10(10):e0138570. doi: 10.1371/journal.pone.0138570 (PMC4595078; doi:10.1371/journal.pone.0138570)
Supplement: S2 Text — (DOCX) [file pone.0138570.s003.docx]

**S2 Text. Sensitivity analyses**

Two main potential biases could affect our estimates of temporal variation in asthma incidence. The first source of bias may be related to the retrospective approach of our estimates. Indeed, only for 3692 subjects (10·4%) asthma incidence was estimated prospectively, while for the majority it was estimated retrospectively. The second one refers to the distortion that could be introduced by the recall of the time at onset of asthma from subjects of different age.

***A.*** *Prospective vs. Retrospective estimates*

**Methods:**

In a subsample of subjects (n=3692) who were surveyed both in 1998-2000 (baseline: BL) and in 2007-2008 (follow-up: FU) screenings, we estimated the rates of asthma incidence between the two surveys using two different approaches, in order to evaluate the bias introduced by measuring asthma incidence with a retrospective compared to a prospective approach.

In the first approach (retrospective), the subjects were classified only on the basis of the FU questionnaire. New cases of asthma were considered those reporting the onset of asthma after the date of filling of the BL questionnaire, while the subjects who reported to have had their first attack of asthma before the BL survey were excluded from the analysis. In the second approach (prospective), the population at risk was defined on the basis of the BL questionnaire, and all the subjects who reported to have had an asthma attack at the BL questionnaire were excluded from the analysis. New cases of asthma were considered all the subjects who reported to have had an attack of asthma at the FU questionnaire, but not at the BL questionnaire.

In both approaches, the time at risk was calculated as the period between the BL and the FU survey for the subjects who didn’t report asthma, or the time between the BL survey and the first attack of asthma for the subjects who had the onset between the baseline and the follow-up survey. The rates of asthma incidence were computed as the ratio between the cases of asthma and the total time at risk. The hazard ratios (HR) for the potential risk factors were estimated using Cox regression models.

**Results**

The rates of asthma incidence were similar when estimated by a retrospective and by a prospective approach (1·93 and 1·70 per 1,000 person-years, respectively, p=0·579), and there were no significant differences in any of the associations with the considered determinants.(**Table3s**)

**Table A. Asthma incidence rates and asthma determinants estimated by retrospective vs. prospective analyses.**

|  | Retrospective approach | Prospective approach | p-value |
| --- | --- | --- | --- |
| Asthma incidence rate† | 1·93 (1·48-2·52) | 1·70 (1·28-2·26) | 0·579 |
| Risk Factors‡ |  |  |  |
| Female sex | 0·90 (0·53-1·54) | 0·76 (0·43-1·35) | 0·672 |
| Birth cohort (ref.1970-9) | 1 (-) | 1 (-) | - |
| 1960-1969 | 1·41 (0·74-2·69) | 1·27 (0·64-2·51) | 0·829 |
| 1947-1959 | 0·62 (0·27-1·43) | 0·65 (0·28-1·52) | 0·934 |
| Hay fever | 6·90 (3·88-12·3) | 5·98 (3·30-10·8) | 0·734 |

†per 1,000 person-years (95%CI); ‡Hazard Ratio (95%CI)

***B.*** *Recall bias evaluation*

As part of a sensitivity analysis to evaluate the effects of recall bias, we compared the models for asthma incidence performed in all subjects (aged 20-84) with models estimated in subjects aged 20-44 at the time of the interview. In fact, in the latter group the extent of the bias can be assumed homogeneous. The age-specific rates of asthma incidence and the effects of period and cohort were almost equivalent in the two analyses (**Figure A**), suggesting that the bias due to a differential recall of the age at the onset of asthma might influence our results to a minor extent.

Table B shows the association of the remission rates of asthma with the potential determinants of remission in the subjects of age 20-44, taken as as sub-group where the recall can be assumed to be homogenous. The results show that there are no significant difference with the main analysis.

**Figure A. Age-period-cohort influences on asthma incidence trends estimated in subjects aged 20-44 (green lines) and in the whole sample (grey lines),** Age-specific rates refers to the reference period (1975). **Fig2S.A** represents the age-specific incidence rates, referred to the reference period 1975; **Fig2S.B** represents the merged period and drift effects (rate ratios), with linear increasing trend determined by the drift, and deviations from linearity (curvature) determined by the period effect; **Fig2S.C** represents the birth cohort effect. The respective regions surrounding the lines provide the 95% confidence intervals (95%CI). Overlapping 95%CI are highlighted with lighter areas.


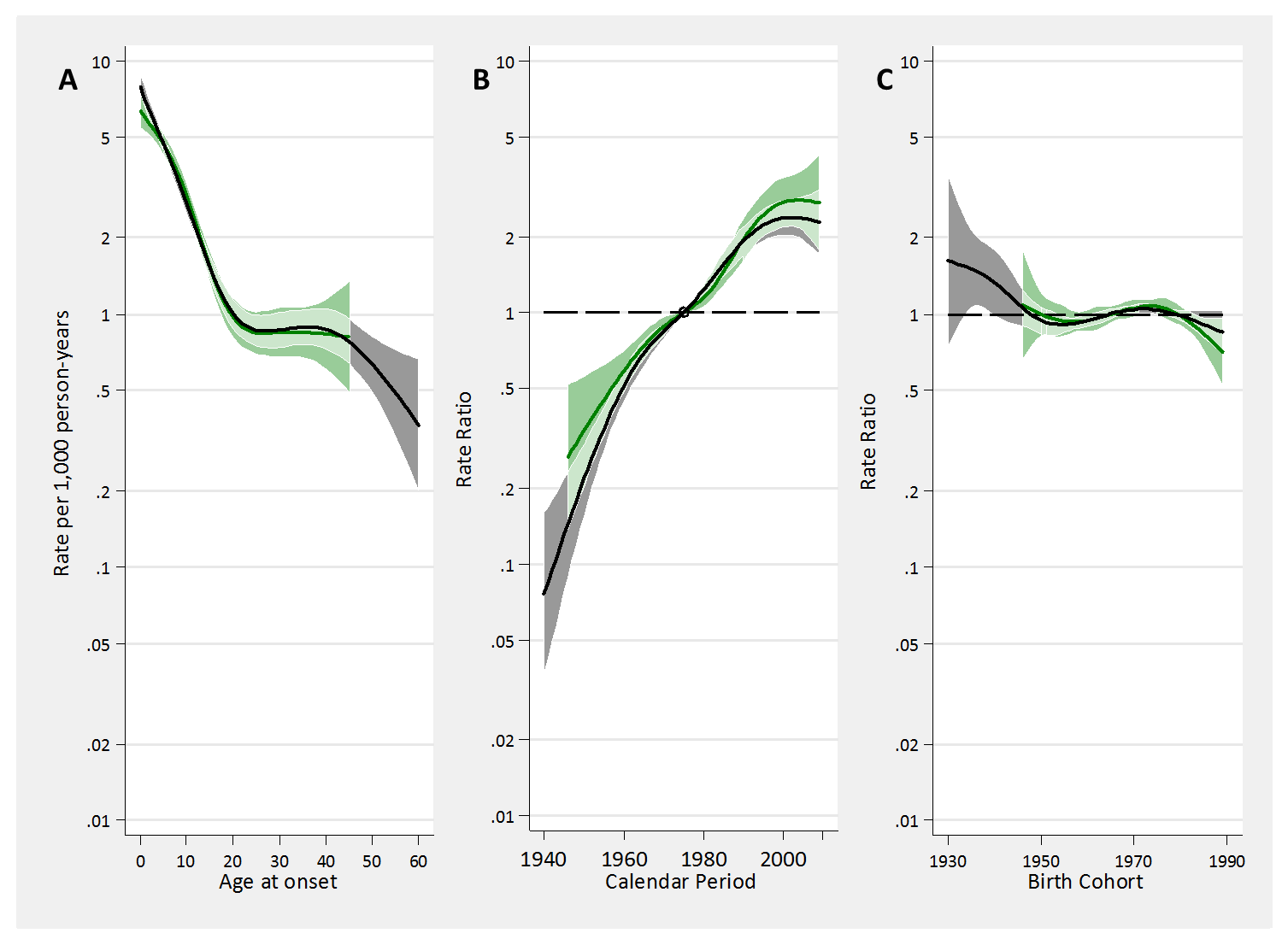


**Table B.** Person-years, number of subjects reporting asthma remission, and crude incidence (95%CI) of remission by sex, presence of hay fever, age at onset, birth cohort, and time since onset, and risk ratios (RR, with 95%CI) for associations with asthma remission in subjects aged 20-44 at the time of the interview.

|  | Subjects  n | | Remissions  n (%) | Time at risk  Person-years | remissions/  1000py  (95% C.I.) | adjusted* RR  (95%CI) |
| --- | --- | --- | --- | --- | --- | --- |
| Sex | |  |  |  |  |  |
| Men | | 1366 | 794 (58·1) | 15566 | 51·0 (47·6-54·7) | 1 (-) |
| Women | | 1266 | 606 (47·9) | 14026 | 43·2 (39·9-46·8) | 0·89 (0·80-0·99) |
| Hay fever | |  |  |  |  |  |
| No | | 998 | 676 (67·7) | 8584 | 78·7 (47·6-84·9) | 1 (-) |
| Yes | | 1613 | 709 (44·0) | 20828 | 34·0 (31·6-36·6) | 0·47 (0·42-0·52) |
| Birth cohort | |  |  |  |  |  |
| before 1959 | | 244 | 134 (54·9) | 2720 | 49·3 (41·6-58·3) | 1 (-) |
| 1960-1969 | | 1051 | 558 (53·1) | 12953 | 43·1 (39·6-46·8) | 0·86 (0·71-1·04) |
| after 1970 | | 1337 | 708 (52·9) | 13918 | 50·9 (47·3-54·8) | 0·85 (0·70-1·03) |
| Age at onset (years) | |  |  |  |  |  |
| 0-14 | | 1762 | 1145 (65·0) | 23182 | 49·4 (46·6-52·3) | 1 (-) |
| 15-29 | | 658 | 223 (34·9) | 5626 | 39·6 (34·8-45·2) | 0·73 (0·62-0·83) |
| 30+ | | 212 | 32 (15·1) | 784 | 40·8 (28·9-57·7) | 0·56 (0·39-0·80) |
| Time since the onset (years) | |  |  |  |  |  |
| 0-9 | | 2632 | 1112 (42.2) | 17347 | 64·1 (60·4-68·0) | 1 (-) |
| 10-19 | | 1120 | 233 (20.8) | 8111 | 28·7 (25·3-32·7) | 0.46 (0.40-0.53) |
| 20-29 | | 532 | 47 (8.8) | 3297 | 14·3 (10·7-19·0) | 0·22 (0·16-0·30) |
| 30+ | | 176 | 8 (4.5) | 836 | 9·6 (4·8-19·1) | 0·14 (0·07-0·28) |
| TOTAL | | **2632** | **1400** | **29592** | **47·3 (44·9-49·9)** |  |
| *RR were adjusted for all the variable included in the table | | | | | | |
