# Supplementary material for: Seventy Years of Asthma in Italy: Age, Period and Cohort Effects on Incidence and Remission of Self-Reported Asthma from 1940 to 2010
Source: PLoS One. 2015 Oct 6;10(10):e0138570. doi: 10.1371/journal.pone.0138570 (PMC4595078; doi:10.1371/journal.pone.0138570)
Supplement: S3 Text — (DOCX) [file pone.0138570.s004.docx]

**S3 Text. Estimates of age-period specific rates of asthma between 1940-2010**

**Methods**

Age classes and calendar period were modeled in 10-years time-intervals. Age-period specific rates of asthma incidence were calculated by using generalized linear model with a Poisson family error structure and an offset of ln*(population at risk)*.

**Results**

In the last 30 years the incidence rates of asthma have nearly doubled and they have nearly decupled since the 1940s. (**Table2s on the online repository**).

**Table A. Estimates of age-specific incidence rates of asthma (per 1,000 person-years, with 95%CI) for each age group.**

| Calendar period | Age group | | | | | | |
| --- | --- | --- | --- | --- | --- | --- | --- |
|  | **0-9** | **10-9** | **20-9** | **30-9** | **40-9** | **50-9** | **60-9** |
| 1930-9 | 0.57  (0.14-2.30) | 0.22  (0.05-0.87) | 0.13  (0.03-0.51) | 0.14  (0.03-0.55) | 0.12  (0.03-0.48) | 0.10  (0.02-0.38) | 0.06  (0.02-0.26) |
| 1940-9 | 1.02  (0.55-1.9) | 0.39  (0.21-0.72) | 0.23  (0.12-0.42) | 0.24  (0.13-0.45) | 0.21  (0.11-0.4) | 0.17  (0.09-0.32) | 0.11  (0.06-0.21) |
| 1950-9 | 2.16  (1.74-2.68) | 0.82  (0.66-1.02) | 0.48  (0.39-0.60) | 0.51  (0.42-0.64) | 0.45  (0.37-0.56) | 0.36  (0.29-0.45) | 0.24  (0.2-0.3) |
| 1960-9 | 3.67  (3.28-4.11) | 1.40  (1.25-1.56) | 0.82  (0.73-0.92) | 0.87  (0.78-0.98) | 0.77  (0.69-0.86) | 0.61  (0.55-0.69) | 0.41  (0.37-0.46) |
| 1970-9 | 4.91  (4.58-5.27) | 1.87  (1.70-2.06) | 1.10  (0.96-1.24) | 1.17  (1.01-1.36) | 1.03  (0.84-1.27) | 0.82  (0.58-1.16) | 0.55  (0.29-1.04) |
| 1980-9 | 6.78  (6.15-7.47) | 2.58  (2.34-2.84) | 1.51  (1.37-1.67) | 1.61  (1.46-1.78) | 1.42  (1.29-1.57) | 1.13  (1.02-1.25) | 0.76  (0.69-0.84) |
| 1990-9 | 7.91  (6.99-8.96) | 3.01  (2.66-3.40) | 1.76  (1.56-2.00) | 1.88  (1.66-2.13) | 1.66  (1.47-1.88) | 1.32  (1.17-1.49) | 0.89  (0.79-1.01) |
| 2000-10 | 9.16  (7.72-10.9) | 3.48  (2.93-4.13) | 2.04  (1.72-2.42) | 2.18  (1.83-2.58) | 1.92  (1.62-2.28) | 1.53  (1.29-1.81) | 1.03  (0.87-1.22) |
